# A hierarchical and configurational analysis of Health Technology Assessment outcomes for cell and gene therapies

**DOI:** 10.3389/fphar.2025.1695961

**Published:** 2025-12-01

**Authors:** Ziyad S. Almalki, Renad M. Alshammari, Nadia A. Dahduli, Mouaddh Abdulmalik Nagi, Syeda Juweria, Moayad M. Alhamdani, Yazan M. Alzahrani, Saja H. Almazrou, Nehad Jaser Ahmed, Abdullah K. Alahmari, Areej A. Alshlowi

**Affiliations:** 1 Department of Clinical Pharmacy, College of Pharmacy, Prince Sattam Bin Abdulaziz University, Al-Kharj/Riyadh, Saudi Arabia; 2 Department of Clinical Pharmacy, College of Pharmacy, Almaarefa University, Ad Diriyah, Saudi Arabia; 3 Department of Pharmacy, Faculty of Medical Sciences, Aljanad University for Science and Technology, Taiz, Yemen; 4 Department of Clinical Pharmacy, College of Pharmacy, King Saud University, Riyadh, Saudi Arabia

**Keywords:** cell and gene therapies, decision-making, Health Technology Assessment, reimbursement, value

## Abstract

**Background:**

Cell and gene therapies (CGTs) challenge traditional Health Technology Assessment (HTA), creating a fragmented global access landscape. This study identifies the determinants of CGT reimbursement outcomes by quantifying the influence of key variables and identifying the configurations leading to a positive recommendation.

**Methods:**

A dual-methodology approach was employed. We constructed a comprehensive dataset of all HTA decisions for CGTs across seven major jurisdictions between January 2017 and July 2025. Hierarchical Linear Modeling (HLM) was used to identify independent predictors of HTA outcomes, and Fuzzy-Set Qualitative Comparative Analysis (fsQCA) was used to identify sufficient pathways to success. Novel composite indicators were developed to measure system-level adaptability and the influence of patient advocacy groups (PAGs).

**Results:**

The HLM analysis, accounting for data clustering (Intraclass Correlation Coefficients (ICCs): 42% country-level, 24% agency-level variance), confirmed that strong clinical efficacy (Coef. = 0.40), high unmet need, and disease rarity were significant positive predictors. High therapy cost was a powerful negative predictor (Coef. = −0.29 per $1M USD). Crucially, high System Adaptability (Coef. = 0.35) and strong PAG Influence (Coef. = 0.28) emerged as major positive determinants. The fsQCA revealed three distinct pathways to a positive recommendation with high consistency: a “Transformative Value” path (consistency: 0.93), a “Strategic Mitigation” path (consistency: 0.90), and an “Economic Dominance” path (consistency: 0.94). The overall QCA solution explained a majority of positive outcomes (solution coverage: 0.68).

**Conclusion:**

HTA success for CGTs is not determined by isolated attributes but by the strategic alignment of therapy-level evidence, agency-level processes, and country-level context. The influence of organized patient advocacy and the structural flexibility of HTA systems are critical, previously under-quantified components of this alignment.

## Introduction

The advent of cell and gene therapies (CGTs) represents a fundamental paradigm shift in medicine (Jian et al., 2025). By correcting the underlying causes of disease at a genetic or cellular level, these technologies offer the potential for durable, and often potentially curative, outcomes from a single administration (Qie et al., 2025; Chancellor et al., 2023). This model stands in stark contrast to the decades-long paradigm of chronic disease management with conventional pharmaceuticals (Qie et al., 2025; Nikitin et al., 2023; Delisle et al., 2025). However, this unprecedented clinical promise has created an access paradox: these transformative “living drugs” are confronting a global reimbursement landscape that is fragmented, unpredictable, and structurally misaligned with their therapeutic and financial model (Phares et al., 2024). This misalignment creates a critical “fourth hurdle” to patient access following regulatory approval for safety, quality, and efficacy (Tsurumaki et al., 2025).

Health Technology Assessment (HTA) is a multidisciplinary process that systematically evaluates the clinical, economic, social, and ethical implications of a health technology (O’Rourke et al., 2020). HTA bodies use this process to inform reimbursement and coverage decisions. However, the unique properties of CGTs creates significant friction with these traditional HTA frameworks. At the core is a structural mismatch between the potentially curative, capital investment paradigm of CGTs and health systems designed for chronic care (Phares et al., 2024; Tsurumaki et al., 2025). The high, one-time upfront cost, which can exceed several million dollars per patient, clashes directly with health system budgets structured around annual, operational expenditures, creating an immediate affordability crisis for payers (Qie et al., 2025; Wagner et al., 2024). This financial pressure is intensified by the limitations of conventional value assessment frameworks (Salzman et al., 2018). The standard cost-per-Quality-Adjusted-Life-Year (QALY) metric struggles to capture the full, lifelong, and societal value of curative therapies, prompting calls to broaden the definition of value (Salzman et al., 2018; Garrison et al., 2021). This limitation is particularly acute for rare genetic diseases, where traditional HTA frameworks that focus on direct healthcare costs inadequately capture the full disease burden (Salzman et al., 2018; Garrison et al., 2023). For instance, a scoping review by Marshall et al. (2023) revealed significant gaps in how economic evaluations assess this burden compared to cost-of-illness studies. The analysis demonstrated that while 82% of cost-of-illness studies adopted a societal perspective, only 15% of economic evaluations did. This resulted in economic evaluations systematically missing key cost categories: productivity losses (10% vs. 77%), travel costs (6% vs. 68%), and family impacts (0% vs. 50%). These findings suggest that current HTA practices may systematically undervalue rare disease interventions by failing to capture the “hidden burden” borne by patients, families, and society, which has important implications for resource allocation decisions. This underscores the need for HTA methodological reform to better accommodate these therapies (Marshall et al., 2023). Furthermore, standard economic conventions, particularly the use of a 3%–5% discount rate, systematically devalue the long-term health gains that are the hallmark of CGTs (Qiu et al., 2024).

This immense financial pressure directly amplifies the scrutiny placed upon a CGT’s evidentiary package. Regulators, often focusing on high unmet need, may grant marketing authorization based on promising but immature data from small, single-arm clinical trials using surrogate endpoints (Tsurumaki et al., 2025). This stands in stark contrast to the traditional preference of HTA bodies for robust comparative evidence from large-scale Randomized Controlled Trials (RCTs), creating a primary source of decision-making uncertainty for payers (Salzman et al., 2018; Garrison et al., 2021).

This complex environment is on the cusp of a seismic shift with the implementation of the EU HTA Regulation (HTAR) 2021/2282, which became applicable from January 2025 for oncology drugs and Advanced Therapy Medicinal Products (ATMPs) (Desmet et al., 2024). The HTAR mandates a single, EU-level Joint Clinical Assessment (JCA) for all new centrally authorized medicines, creating a harmonized report on clinical effectiveness and safety (Desmet et al., 2024). However, crucial non-clinical aspects, including economic evaluation, budget impact analysis, and final reimbursement and pricing decisions, will remain outside the jurisdiction of the JCA, becoming the exclusive competence of individual member states (Jakubowski et al., 2024). This bifurcation of the assessment process makes the present study particularly timely. As the JCA standardizes the evaluation of the clinical evidence for the ATMP, the national-level factors this study investigates—such as economic capacity, the flexibility of the HTA system, and stakeholder influence—will become the primary locus of differentiation and the key determinants of whether a positive EU-level clinical assessment translates into actual patient access on the ground (Jakubowski et al., 2024).

While the existing literature provides a rich description of the problems facing CGTs (Phares et al., 2024; Tsurumaki et al., 2025; Wagner et al., 2024; Salzman et al., 2018; Garrison et al., 2021), it lacks a systematic, multi-country explanation of how these factors combine to produce HTA outcomes. The field is dominated by single-country case studies (Delisle et al., 2025; Phares et al., 2024) and qualitative analyses that, while valuable, lack the generalizability and predictive power needed for robust strategic insights. This study moves beyond problem description to causal explanation and configurational modeling. It employs a novel dual-methodology to identify not only the key independent determinants of HTA outcomes but also the specific configurations of factors that are sufficient for achieving reimbursement. Therefore, this study addresses these gaps by quantifying the influence of therapy-, agency-, and country-level variables on the probability of HTA recommendations for CGTs across multiple countries, identify the necessary and sufficient configurations of these variables that lead to positive HTA recommendations, and synthesize the findings from this dual-methodology into an actionable strategic framework for stakeholders. This framework is designed to help stakeholders better navigate the complex CGT reimbursement landscape. By providing a clear and strategic guide, the study hopes to enhance the capability of stakeholders to achieve positive HTA outcomes and secure the necessary reimbursements for CGTs. We acknowledge that this framework provides a strategic guide for stakeholders (primarily developers, patient groups, and policymakers) to navigate the reimbursement process; it does not imply that all therapies will, or should, be deemed ‘value for money’ based on national criteria.

## Methods

### Study design and country selection

This study employs a synergistic dual-methodology approach, combining Hierarchical Linear Modeling (HLM) and Fuzzy-Set Qualitative Comparative Analysis (fsQCA) to analyze HTA decisions. This dual-methodology is necessary because the two methods answer different, complementary questions. HLM is a variable-based approach that identifies the net effect of individual predictors (e.g., “what is the independent impact of cost?”). In contrast, fsQCA is a case-based, configurational approach that identifies which combinations of factors are sufficient for success (e.g., “what “recipes” lead to a positive recommendation?”). Using both provides a more comprehensive explanation of HTA decision-making.

The analysis focuses on seven key HTA jurisdictions: Canada: Canadian Agency for Drugs and Technologies in Health (CADTH); United Kingdom: National Institute for Health and Care Excellence (NICE); Germany: The Federal Joint Committee (Gemeinsamer Bundesausschuss, G-BA); France: The French National Authority for Health (Haute Autorité de Santé, HAS); Italy: The Italian Medicines Agency (Agenzia Italiana del Farmaco, AIFA); Spain: The Spanish Agency of Medicines and Medical Devices (Agencia Española de Medicamentos y Productos Sanitarios, AEMPS); Australia: Pharmaceutical Benefits Advisory Committee (PBAC) and Medical Services Advisory Committee (MSAC). These jurisdictions were selected to represent a diverse range of mature HTA systems with transparent, public processes (Fontrier et al., 2022). This heterogeneity allows for the robust testing of our hypotheses across different value assessment paradigms, including those with formal cost-per-QALY frameworks (e.g., NICE in the United Kingdom, CADTH in Canada, PBAC in Australia), those focused on “added clinical value” (e.g., G-BA in Germany, HAS in France), and those with hybrid or distinct models (e.g., AIFA in Italy, AEMPS in Spain) (Fontrier et al., 2022; Drummond et al., 2022). The study was guided by three core hypotheses: (H1) That HTA outcomes are determined by a combination of therapy-level (e.g., clinical efficacy, cost), agency-level (e.g., assessment framework, adaptability), and country-level (e.g., GDP) factors; (H2) That agency- and country-level factors will explain a significant portion of the variance in outcomes, validating a multi-level approach; and (H3) That there is no single “silver bullet” factor, but rather multiple, configuration-based pathways (equifinality) to a positive HTA recommendation.

### Data and sample

To conduct the analysis, we first identified all published HTA reports for CGTs from the selected agencies, and the data contained within these reports were used to construct a comprehensive dataset. The study period (1 Jan 2017 - 31 July 2025) was chosen to capture the entire modern cohort of transformative CGTs (e.g., Kymriah, Yescarta, Luxturna) that have challenged HTA systems. Earlier isolated approvals, such as Glybera (MA 2012, withdrawn 2017), were excluded as they are not representative of the current wave of ATMPs and their associated HTA paradigms.

The search strategy involved a targeted review of the official websites of the seven selected HTA bodies, supplemented by a systematic search of two electronic databases: MEDLINE (via PubMed), and the International Network of Agencies for Health Technology Assessment (INAHTA) database. This was done to identify any reviews missed in the primary search and to locate related publications. The search strategy combined a comprehensive list of all FDA- and EMA-approved CGTs (both brand and generic names) with a set of validated HTA-related keywords. The full, unabridged search strategy, including database search strings and manual review protocol, is provided in Supplementary Appendix A.

To ensure the search was exhaustive, the reference lists of all included HTA reports and relevant systematic reviews were manually searched (a “snowballing” technique) to identify additional potential reviews. For every HTA review that met the inclusion criteria, all publicly available documentation was systematically downloaded and archived. This included, but was not limited to, the final recommendation or guidance document, committee meeting minutes, manufacturer’s evidence submission, patient and clinician group submissions, and any related economic modeling reports.

An HTA appraisal was included if it met four criteria (Jian et al., 2025): the product was an FDA- or EMA-defined CGT (Qie et al., 2025); the therapy had received marketing authorization (Chancellor et al., 2023); the review was conducted by one of the seven specified HTA bodies and concluded within the study timeframe; and (Nikitin et al., 2023) a final, public recommendation document with rationale was available. Importantly, inclusion was not contingent on a positive outcome; therapies that received a negative recommendation or were subsequently withdrawn from the market (e.g., Zynteglo in Germany, see Table 1) were included in the analysis. Reviews with insufficient public documentation were excluded.

**TABLE 1:** Cohort of included cell and gene therapies and HTA submissions (2017–2025).

Therapy name (generic)	Indication	Manufacturer	HTA agency	Country	HTA submission year	Final HTA outcome	Key nuances of outcome	Standardized outcome score (+2 to −2)
Kymriah (tisagenlecleucel)	ALL	Novartis	NICE	United Kingdom	2018	Recommended	Initially via cancer drugs fund, now routine use	+1
Kymriah (tisagenlecleucel)	DLBCL	Novartis	NICE	United Kingdom	2023	Terminated appraisal	Terminated by manufacturer due to lack of cost-effectiveness	−2
Kymriah (tisagenlecleucel)	ALL	Novartis	CADTH	Canada	2018	Reimburse with conditions	Clinical criteria and conditions apply	0
Kymriah (tisagenlecleucel)	DLBCL, ALL	Novartis	G-BA	Germany	2024	Hint for non-quantifiable benefit	Reassessment; orphan status benefit assumed but not quantifiable	0
Kymriah (tisagenlecleucel)	DLBCL, ALL	Novartis	HAS	France	2019	Important SMR/ASMR III	Favorable opinion for reimbursement	0
Kymriah (tisagenlecleucel)	DLBCL, ALL	Novartis	AIFA	Italy	2019	Reimbursed	Payment at results (PaR) model; innovation status	0
Yescarta (axicabtagene ciloleucel)	DLBCL, HGBL	Kite/Gilead	NICE	United Kingdom	2018	Recommended	Available via cancer drugs fund^20^	0
Yescarta (axicabtagene ciloleucel)	DLBCL, HGBL	Kite/Gilead	CADTH	Canada	2023	Reimburse with conditions	Price reduction required	0
Yescarta (axicabtagene ciloleucel)	DLBCL, HGBL	Kite/Gilead	G-BA	Germany	2024	Hint for non-quantifiable benefit	Reassessment after deadline	0
Yescarta (axicabtagene ciloleucel)	DLBCL, PMBCL	Kite/Gilead	HAS	France	2019	Reimbursed 3rd Line+	ASMR III (DLBCL)/ASMR V (PMBCL)	0
Yescarta (axicabtagene ciloleucel)	DLBCL, PMBCL	Kite/Gilead	AIFA	Italy	2019	Reimbursed	Payment at results (PaR) model	0
Luxturna (voretigene neparvovec)	Inherited retinal dystrophy	Spark/Novartis	NICE	United Kingdom	2019	Recommended	Commercial arrangement in place	+1
Luxturna (voretigene neparvovec)	Inherited retinal dystrophy	Spark/Novartis	G-BA	Germany	2019	Considerable benefit	Positive recommendation	+1
Luxturna (voretigene neparvovec)	Inherited retinal dystrophy	Spark/Novartis	HAS	France	2024	Important SMR/ASMR II	Favorable opinion for reimbursement	+1
Luxturna (voretigene neparvovec)	Inherited retinal dystrophy	Spark/Novartis	AIFA	Italy	2021	Reimbursed via innovative fund	Innovation status granted	+2
Luxturna (voretigene neparvovec)	Inherited retinal dystrophy	Spark/Novartis	PBAC	Australia	2020	Recommended with MEA	Outcomes-based payment agreement	0
Luxturna (voretigene neparvovec)	Inherited retinal dystrophy	Spark/Novartis	CADTH	Canada	2020	Reimburse with conditions	Uncertainty in long-term efficacy	0
Zolgensma (onasemnogene abeparvovec)	Spinal muscular atrophy	AveXis/Novartis	NICE	United Kingdom	2021	Recommended	Landmark deal involving patient groups	+1
Zolgensma (onasemnogene abeparvovec)	Spinal muscular atrophy	AveXis/Novartis	G-BA	Germany	2021	No added benefit proven	Does not block access for orphans but weakens price negotiation	−1
Zolgensma (onasemnogene abeparvovec)	Spinal muscular atrophy	AveXis/Novartis	PBAC	Australia	2021	Deferred	Deferred multiple times before eventual funding	−1
Zynteglo (betibeglogene autotemcel)	Beta-thalassemia	Bluebird bio	G-BA	Germany	2019	Unquantifiable benefit	Manufacturer later withdrew from market over price negotiations	−2
Tecartus (brexucabtagene autoleucel)	ALL	Kite/Gilead	NICE	United Kingdom	2023	Recommended	Available via cancer drugs fund^34^	0
Tecartus (brexucabtagene autoleucel)	MCL	Kite/Gilead	CADTH	Canada	2021	Reimburse with conditions	Price reduction required	0
Tecartus (brexucabtagene autoleucel)	MCL, ALL	Kite/Gilead	G-BA	Germany	2021	Hint for non-quantifiable benefit	Orphan drug status	0
Tecartus (brexucabtagene autoleucel)	MCL	Kite/Gilead	HAS	France	2024	Substantial SMR/ASMR minor	Reassessment with real-world data	0
Abecma (idecabtagene vicleucel)	Multiple myeloma	BMS/bluebird bio	CADTH	Canada	2021	Do not reimburse	Negative recommendation	−2
Abecma (idecabtagene vicleucel)	Multiple myeloma	BMS/bluebird bio	NICE	United Kingdom	2025	Terminated appraisal	Terminated by manufacturer; no evidence submitted	−2
Abecma (idecabtagene vicleucel)	Multiple myeloma	BMS/bluebird bio	AIFA	Italy	2022	Reimbursed class H	Reimbursed for hospital use with expert supervision	0
Carvykti (ciltacabtagene autoleucel)	Multiple myeloma	Janssen/Legend	CADTH	Canada	2024	Reimburse with conditions	Price reduction required due to high ICER	0
Carvykti (ciltacabtagene autoleucel)	Multiple myeloma	Janssen/Legend	G-BA	Germany	2024	Hint of non-quantifiable benefit	For patients with 1–3 prior therapies	0
Carvykti (ciltacabtagene autoleucel)	Multiple myeloma	Janssen/Legend	MSAC	Australia	2024	Deferred	Deferred multiple times; clinical value accepted but price rejected	−1
Hemgenix (etranacogene dezaparvovec)	Hemophilia B	uniQure/CSL behring	G-BA	Germany	2023	Non-quantifiable benefit	Single-arm trial data assessed	0
Hemgenix (etranacogene dezaparvovec)	Hemophilia B	uniQure/CSL behring	CADTH	Canada	2023	Reimburse with conditions	Price reduction required; long-term uncertainty	0
Hemgenix (etranacogene dezaparvovec)	Hemophilia B	uniQure/CSL behring	NICE	United Kingdom	2024	Recommended via managed access	Conditional approval for further data collection	0
Hemgenix (etranacogene dezaparvovec)	Hemophilia B	uniQure/CSL behring	AIFA	Italy	2025	Reimbursed	Approved for reimbursement by AIFA board	+1
Libmeldy (atidarsagene autotemcel)	Metachromatic leukodystrophy	Orchard therapeutics	NICE	United Kingdom	2022	Recommended	Recommended via highly specialised technologies (HST) pathway	+1
Libmeldy (atidarsagene autotemcel)	Metachromatic leukodystrophy	Orchard therapeutics	G-BA	Germany	2021	Major/Non-quantifiable benefit	Split rating based on patient symptom status	0
Libmeldy (atidarsagene autotemcel)	Metachromatic leukodystrophy	Orchard therapeutics	HAS	France	2023	Negotiations ended	Initial split recommendation, but price negotiations failed	−2
Libmeldy (atidarsagene autotemcel)	Metachromatic leukodystrophy	Orchard therapeutics	AIFA	Italy	2022	Reimbursed	Full reimbursement approved	+1
Casgevy (exagamglogene autotemcel)	Beta thalassemia, SCD	Vertex	CADTH	Canada	2024	Reimburse with conditions	Reimbursement recommended with conditions	0
Casgevy (exagamglogene autotemcel)	Beta thalassemia, SCD	Vertex	NICE	United Kingdom	2025	Recommended via managed access	Conditional approval	0
Casgevy (exagamglogene autotemcel)	Beta thalassemia, SCD	Vertex	G-BA	Germany	2025	Hint for non-quantifiable benefit	Orphan drug status	0
Lyfgenia (lovotibeglogene autotemcel)	Sickle cell disease	Bluebird bio	CADTH	Canada	2025	Reimburse with conditions	Reimbursement recommended with conditions	0
Roctavian (valoctocogene roxaparvovec)	Hemophilia A	BioMarin	G-BA	Germany	2023	Additional benefit proven (orphan)	Supply restricted to providers in data collection registry	0
Roctavian (valoctocogene roxaparvovec)	Hemophilia A	BioMarin	NICE	United Kingdom	2025	Recommended	Positive recommendation	+1
Upstaza (eladocagene exuparvovec)	AADC deficiency	PTC therapeutics	NICE	United Kingdom	2023	Recommended	Transformative benefits noted; commercial deal in place	+1
Upstaza (eladocagene exuparvovec)	AADC deficiency	PTC therapeutics	AIFA	Italy	2023	Reimbursed class H	Reimbursed for hospital use with expert supervision	0
Upstaza (eladocagene exuparvovec)	AADC deficiency	PTC therapeutics	HAS	France	2023	ASMR III/Moderate CAV	Moderate clinical added value assigned	0

### Data extraction and verification process

To ensure the reliability and validity of the dataset, a rigorous, dual-reviewer data extraction process was implemented. The variables for extraction were selected based on a theoretical framework of HTA decision-making. While we referenced the domains of the EUnetHTA Core Model, version 3, a custom data extraction form was necessary. The Core Model domains (e.g., “Organizational Aspects,” “Patients and Social aspects”) are designed for comprehensive qualitative assessment, whereas our dual-methodology required the development of more granular, quantifiable variables (e.g., “System Adaptability,” “PAG Influence,” see Table 2 and Appendix B) capable of being used in HLM and QCA models. Data from all source documents for each included HTA review were extracted in parallel by two independent researchers. Following the independent extraction, the two resulting datasets were programmatically compared to identify any discrepancies. Disagreements were resolved through a consensus-based process. The two primary reviewers would first meet to re-examine the source documents and discuss the conflicting entries. If an agreement could not be reached, the discrepancy was escalated to a third, senior reviewer for a final, binding decision. This multi-step process was designed to minimize error and ensure the consistency of the final dataset used for the HLM and QCA.

**TABLE 2:** Definition, operationalization, and data sources for study variables.

Level	Variable name	Definition	How measured/coded	Data source(s)
Therapy	Clinical efficacy	The demonstrated clinical benefit of the therapy in pivotal trials	Coded based on primary endpoint: 1 = Superiority on final outcome (e.g., OS); 0 = Superiority on validated surrogate; −1 = Superiority on unvalidated surrogate	HTA reports, EPARs, FDA reviews
	Comparative evidence	The design of the pivotal clinical trial(s)	Categorical: 1 = Randomized controlled trial (RCT); 0 = Single-Arm trial with matched external control; −1 = Single-Arm trial, no formal comparator	HTA reports, ClinicalTrials.gov
	Safety profile	The severity and frequency of adverse events	Ordinal scale (1–5) based on rate of grade 3+ AEs and presence of black box warnings	HTA reports, product labels
	Therapy cost	The list price or wholesale acquisition cost per treatment course	Continuous variable (in USD, PPP-adjusted). For QCA, calibrated with anchors based on ICER thresholds (e.g., <$1M, $2.5M, >$4M)	HTA reports, press releases
	Unmet Need^a^	The severity of the disease and lack of effective alternative treatments	Composite score (1–5) based on disease mortality/morbidity and HTA body’s explicit statement on availability of alternatives	HTA reports, Published Literature
	Disease Rarity^b^	The prevalence of the target indication	Binary: 1 = orphan/rare disease designation; 0 = Non-orphan	HTA reports, regulatory designations
Agency	Assessment framework	The primary methodology used by the HTA agency for value assessment	Categorical: 1 = Formal cost-per-QALY threshold (e.g., NICE); 0 = added clinical value framework (e.g., G-BA, HAS); −1 = Other/unclear	Agency methodology guidelines
	System adaptability	The agency’s structural capacity to accommodate CGTs, including special pathways and innovative payment models	Composite score (1–5) based on existence of formal flexible pathways (e.g., NICE HST), established frameworks for RWE, and documented use of outcomes-based contracts	Agency methodology guidelines, policy documents, literature
	PAG influence	The documented level of engagement by Patient Advocacy Groups in the specific HTA review	Ordinal: 2 = Active participation in committee meetings; 1 = Formal written submission from PAG; 0 = No documented PAG submission	HTA reports, patient group websites
Country	Economic status (GDP)	The country’s national wealth	GDP *per capita* (constant USD, PPP-adjusted)	World Bank, OECD databases
	Health expenditure	The proportion of national wealth dedicated to healthcare	Total health expenditure as a percentage of GDP.	WHO global health expenditure database, OECD

^a^ This operationalization aligns with major HTA, frameworks, which define ‘unmet need’ as a function of both disease severity and the inadequacy of existing treatments.

^b^ This coding is based on formal regulatory designation, which itself is based on disease prevalence, the standard metric used by bodies like the FDA, and EMA.

### Variable operationalization

To analyze the determinants of HTA outcomes, a multi-level conceptual framework was adopted. A multi-level (hierarchical) model was not just an option but a statistical necessity. Our data are inherently nested: multiple HTA decisions (Level 1) are “clustered” within specific HTA agencies (Level 2), which are themselves “clustered” within countries (Level 3). Analyzing this data with a standard, single-level regression would violate the assumption of independent observations. This non-independence leads to underestimated standard errors and a severely inflated risk of Type I errors (false positives). The HLM approach is the appropriate method to model this data structure correctly, allowing us to partition the variance at each level. This framework recognizes that HTA decisions are nested phenomena, where therapy-specific attributes are evaluated within the context of specific HTA agencies, which are themselves situated within national healthcare systems. This structure necessitates a multi-level approach to account for variance at therapy, agency, and country levels.

The primary outcome variable, the final HTA recommendation, was coded on a five-level ordinal scale to capture the nuanced nature of reimbursement decisions: +2 (Recommended with Innovation Status): A positive recommendation with a formal designation of high innovation, often unlocking special funding pools. +1 (Recommended): An unconditional or straightforward recommendation for reimbursement. 0 (Restricted/Managed Access): A recommendation for reimbursement subject to specific clinical criteria, patient subgroups, or financial/data-generation arrangements (e.g., managed entry agreements, coverage with evidence development). −1 (Negative on Economic Grounds/Deferred): A negative recommendation or deferral where clinical value is acknowledged but the therapy is deemed not cost-effective at the submitted price. −2 (Negative on Clinical Grounds/Terminated/Withdrawn): An outright rejection due to insufficient clinical evidence, or an appraisal terminated by the manufacturer. This scale was developed to ensure consistency and comparability across jurisdictions that use different recommendation frameworks (e.g., QALY vs. Added Benefit). This ordinal approach captures more granular information than a simple binary (yes/no) outcome (e.g., it differentiates a “restricted” from a “full” [+1] recommendation) and has been shown to increase statistical power and analytical nuance. While the precise reason for termination is often not public, it is typically interpreted by HTA bodies and researchers as a withdrawal in anticipation of a negative outcome, and thus coded as such.

Predictor variables were operationalized at three levels. Therapy, agency and country. Each level contains several related items. With the agency level, two novel composite indicators, System Adaptability and patient advocacy groups (PAG) Influence, were constructed to quantify complex systemic and stakeholder factors. The detailed methodology for the construction and validation of these indicators, which adheres to OECD best practices, is provided in Supplementary Appendix B. Full details are provided in Table 2.

### Analytical strategy

#### Hierarchical linear modeling (HLM)

HLM, or mixed-effects modeling, is a statistical technique specifically designed for analyzing nested data structures (Bash et al., 2020). A three-level random-intercept and random-slope model was specified. Analysis was conducted using SAS software, with missing data handled via multiple imputation. We used multiple imputation as it is superior to complete-case analysis, which can introduce significant bias and reduce statistical power if data are not missing completely at random. Our procedure assumed data were missing at random and used a multivariate imputation by chained equations approach. Crucially, the imputation model was designed to respect structural missingness. For example, cost-effectiveness variables (e.g., ICERs) are structurally absent from agencies that do not use them (e.g., G-BA, HAS). These “structural zeros” were not imputed. The multiple imputation algorithm was applied only to data points that were stochastically missing (i.e., a value was expected but not reported). This ensures that our imputed datasets do not create ‘results which lack validity’ and adhere to best practices for handling missing health economic data.

### Fuzzy-set qualitative comparative analysis (fsQCA)

Fuzzy-set Qualitative Comparative Analysis (fsQCA) is a research method that focuses on cases to find which combinations of factors lead to a specific outcome. Think of it like finding different recipes for success. It's particularly useful for understanding complex situations where multiple pathways can lead to the same result (equifinality) and where the effect of one factor depends on the presence of others (conjunctural causation). Instead of isolating single variables, fsQCA identifies which specific “bundles” of conditions are necessary or sufficient to produce the outcome you’re studying. To clarify these distinct concepts: a necessary condition is one that must be present for an outcome to occur (but does not guarantee it), whereas a sufficient condition (or configuration) is one that, if present, guarantees the outcome (but may not be the only way to achieve it). Our study explicitly tests for both.

The analysis unfolds in two main stages. First is Calibration, where raw data is converted into scores ranging from 0 (completely out of a category) to 1 (fully in a category). This isn't just a mechanical step; it requires defining clear, theory-based thresholds. For example, you’d set specific cost points to determine how much a therapy qualifies as a “high-cost therapy.” The second stage is the Truth Table Analysis. As shown in Supplementary Appendix C, a table is built to show every observed combination of conditions and how consistently each one leads to the outcome. Combinations that reliably produce the result (e.g., with a consistency score over 0.80) are then logically simplified using Boolean algebra to reveal the most essential and straightforward recipes for achieving the outcome.

## Results

### Part I: statistical determinants of HTA outcomes (HLM results)

All HLM results presented are the pooled estimates from the 10 multiply imputed datasets, which were combined using Rubin’s Rules. The HLM null model (an intercept-only model used to partition variance) confirmed the appropriateness of a multi-level approach over a standard regression model. The final three-level HLM (Table 3) included all variables identified *a priori* based on HTA theory and literature (see Introduction). A null model was run first to establish the ICCs, followed by the full model presented here. We did not engage in stepwise variable removal, as our goal was explanatory, not predictive. The Intraclass Correlation Coefficients (ICCs), interpreted as variance partition coefficients (VPCs), were substantial (ICC-Country = 0.42; ICC-Agency = 0.24). This indicates that 42% of the total variance in HTA outcomes is attributable to country-level differences and 24% to agency-level differences. The statistical significance of these random-effect variance components (p < 0.001) for both Level 2 and Level 3) confirmed that this clustering is not due to chance. Furthermore, the results showed that at the therapy level, strong clinical efficacy (Coefficient = 0.40, p = 0.006) and comparative evidence from an RCT (Coefficient = 0.33, p = 0.041) were positive predictors. Conversely, therapy cost emerged as a strong negative predictor (Coefficient = −0.29 per $1M USD, p < 0.001), partially offset by high unmet need (Coefficient = 0.31, p = 0.004) and disease rarity (Coefficient = 0.22, p = 0.025). At the agency level, high system adaptability (Coefficient = 0.35, p = 0.019) and PAG influence (Coefficient = 0.28, p = 0.022) were associated with positive recommendations, with patient group involvement proving influential. At the country level, GDP *per capita* was a significant positive predictor (Coefficient = 0.53 per $10k USD, p < 0.001). The model also identified a significant cross-level interaction between therapy cost and GDP *per capita* (Coefficient = 0.06, p = 0.010), indicating that a country’s wealth mitigates the negative impact of high therapy costs on HTA outcomes, thus suggesting that the ability to pay is crucial in value assessment.

**TABLE 3:** Results of the three-level hierarchical linear model predicting HTA outcome score.

Parameter	Coefficient	Std. Error	p-value
Fixed effects			
Intercept (γ000)	−0.92	0.23	<0.001
Level 1: Therapy			
Clinical Efficacy	0.40	0.14	0.006
Comparative Evidence (RCT)	0.33	0.16	0.041
Safety Profile	0.10	0.06	0.031
Therapy Cost (per $1M USD)	−0.29	0.08	<0.001
Unmet Need	0.31	0.11	0.004
Disease Rarity (Orphan)	0.22	0.10	0.025
Level 2: Agency			
Assessment Framework (QALY)	−0.19	0.09	0.042
System Adaptability	0.35	0.15	0.019
PAG Influence	0.28	0.12	0.022
Level 3: Country			
GDP *per capita* (per $10k USD)	0.53	0.15	<0.001
Health Expenditure (% of GDP)	0.21	0.09	0.011
Cross-Level Interaction			
Therapy Cost * GDP *per capita*	0.06	0.02	0.010
Random Effects (Variance Components)			
Level 1 (Residual)	0.26		
Level 2 (Agency)	0.18		
Level 3 (Country)	0.32		
Intraclass Correlation (ICC) - Country	0.42		
Intraclass Correlation (ICC) - Agency	0.24		

This table presents the fixed effects coefficients, standard errors, and p-values from the final HLM model. The model accounts for the nested structure of the data (decisions within agencies, agencies within countries). The dependent variable is the Standardized Outcome Score (+2 to −2). The significant Intraclass Correlation Coefficients (ICCs) confirm that a substantial portion of the variance in outcomes is explained by agency- and country-level differences, validating the use of a multi-level approach.

### Part II: configurational pathways to HTA success (QCA results)

The fsQCA analysis moves beyond the net effects of individual variables to identify combinations of conditions. First, we conducted an analysis of necessity. This analysis confirmed that no single factor was a “silver bullet” (all consistency scores for individual necessary conditions were <0.9), confirming that success is configurational. The analysis of sufficiency, however, revealed three distinct and logically parsimonious pathways to a positive recommendation (Table 4), demonstrating the principle of equifinality.

**TABLE 4:** Summary of Configurational paths to positive HTA outcomes.

Path	Therapy conditions	Agency/Country conditions	Core conditions	Consistency	Raw coverage	Unique coverage
1: Transformative value	High efficacy* High unmet need* Favorable safety	High GDP	High Efficacy * High unmet need * Favorable safety * High GDP	0.93	0.38	0.20
2: Strategic mitigation	Rare disease* High uncertainty	High System Adaptability* Innovative Payment Model Strong PAG Influence	Rare disease * High uncertainty * High system adaptability * Innovative payment model * Strong PAG influence	0.90	0.31	0.15
3: Economic dominance	Superior safety * Cost-saving	∼	Superior safety * Cost-saving	0.94	0.14	0.08
Overall solution				**0.91**	**0.68**	

This table presents the results of the fuzzy-set Qualitative Comparative Analysis (fsQCA). Each row represents a distinct combination of conditions (a “pathway”) that is sufficient for achieving a positive HTA outcome. “Consistency”: Measures the extent to which a pathway reliably leads to the outcome (analogous to ‘significance’). A score of 0.93 means that 93% of cases with that configuration had a positive outcome. “Raw Coverage”: Measures the proportion of all positive outcomes explained by a specific pathway (analogous to ‘effect size’). “Unique Coverage”: Measures the proportion of positive outcomes explained only by that specific pathway. “Overall Solution”: metrics show the explanatory power of all three pathways combined (i.e., The ‘Overall Solution’ consistency (0.91) and coverage (0.68) indicate that the three identified pathways, combined, are highly reliable and explain 68% of all positive HTA outcomes in our dataset).

The analysis for sufficiency, however, revealed three distinct and logically parsimonious pathways to a positive recommendation, demonstrating the principle of equifinality. These pathways, summarized in Table 4, represent different strategic logics for achieving reimbursement.

Pathway 1: The “Transformative Value” Path (Consistency: 0.93, Raw Coverage: 0.38). This pathway describes a “breakthrough” therapy with a combination of High Efficacy, addressing a High Unmet Need, and having a Favorable Safety profile. When presented in a High GDP country, this compelling clinical case is highly likely to receive a positive recommendation.

Pathway 2: The “Strategic Mitigation” Path (Consistency: 0.90, Raw Coverage: 0.31). This pathway illustrates how success can be achieved even with an uncertain evidence base (e.g., based on surrogate endpoints). The key enabling conditions are submission to an agency with High System Adaptability, a proactive offer of an Innovative Payment Model, and critically, Strong PAG Influence. This pathway demonstrates that strategic flexibility, system readiness, and a powerful patient voice can successfully compensate for an immature evidence package.

Pathway 3: The “Economic Dominance” Path (Consistency: 0.94, Raw Coverage: 0.14). This less common pathway is based on a compelling economic argument. It describes a therapy that, in addition to being clinically effective, has a Superior Safety profile and is demonstrably Cost-Saving. In this scenario, a positive recommendation is almost guaranteed, regardless of other contextual factors.

Finally, a parallel fsQCA was conducted for the absence of a positive recommendation (i.e., outcome scores of 0, -1, or -2). This analysis is crucial for testing the principle of causal asymmetry—the concept that the “recipe” for failure is not merely the mirror opposite of the recipe for success. The analysis revealed one highly consistent and parsimonious pathway to a negative outcome (Overall Solution Consistency: 0.91; Overall Solution Coverage: 0.45): Veto Pathway (Consistency: 0.91; Raw Coverage: 0.45): High Evidence Uncertainty * High Therapy Cost * ∼System Adaptability. This “veto pathway” is highly informative. It demonstrates that the combination of high cost and high clinical uncertainty is toxic, particularly when the HTA system itself is rigid (the absence, or “tilde”, of System Adaptability). This finding empirically supports the logic of our “Strategic Mitigation” path (Pathway 2): System Adaptability and Strong PAG Influence are effective precisely because they function as the direct counters to this potent veto configuration.

## Discussion

This study moves beyond descriptive accounts of the challenges facing CGTs to a systematic, multi-country analysis of the determinants of their HTA outcomes. A key conclusion is that HTA success does not derive from a single, dominant factor but from the synergistic combination of clinical, economic, and systemic elements. The HLM identifies the key independent predictors, while the QCA reveals the specific causal configurations through which these factors combine. For instance, the HLM identifies System Adaptability and PAG Influence as key predictors. The “Strategic Mitigation” pathway from our QCA demonstrates precisely how these factors work in concert: they allow HTA systems to grant access even when traditional evidentiary requirements are not met, effectively substituting for evidence with managed risk and stakeholder consensus.

Our quantitative findings resonate strongly with, and provide empirical validation for, the key themes in the contemporaneous 2024–2025 literature. Our HLM analysis, for example, is the first to our knowledge to quantify the independent, positive effect of System Adaptability (Coefficient = 0.35, p = 0.019) on CGT reimbursement outcomes. This finding provides empirical evidence for the arguments made by numerous recent policy analyses (Phares et al., 2024; Hariram and Gavan, 2025) that an agency’s structural capacity to manage uncertainty and implement novel payment models is no longer a “soft” factor, but a hard determinant of access. It reflects the implementation constraints and infrastructure gaps (Hariram and Gavan, 2025) that national systems are currently facing. Similarly, our HLM finding for PAG Influence (Coefficient = 0.28, $p = 0.022) moves beyond case-study-level observations. It provides robust, multi-national quantitative validation that organized patient advocacy is a statistically significant predictor of HTA outcomes, a theme highlighted as central to building trust in innovation and navigating the modern health landscape in recent 2024 analyses (Marin and Hasdeu, 2025).

A core finding is that the context in which a CGT is assessed is as important as its intrinsic characteristics. The HLM identified country-level GDP *per capita* as a powerful positive predictor and a significant moderator that weakens the negative impact of high Therapy Cost. While this suggests a straightforward affordability argument, our analysis points to a more nuanced mechanism we term the “Adaptability Gap.”

The significant positive effect of System Adaptability in the HLM suggests that an agency’s structural capacity to accommodate CGTs through flexible pathways and innovative contracts is a major determinant of success. This adaptability, however, is not simply a matter of policy preference; it requires significant investment in health system infrastructure (Wu et al., 2025; Angelis et al., 2020). The implementation of the very tools that define an adaptable system—such as outcomes-based innovative payment models (IPMs) and real-world evidence (RWE) registries—requires substantial and sustained funding for data systems, analytical capabilities, and administrative expertise (Wu et al., 2025; Shi et al., 2025). A country’s GDP, therefore, likely influences HTA outcomes not just through the direct capacity to absorb high costs, but also by enabling investment in the sophisticated infrastructure required for these flexible arrangements (Iglesias-López et al., 2023; Rejon-Parrilla et al., 2023; Qiu et al., 2022). This infrastructural capacity allows a health system to transition from a rigid cost-containment function to a more flexible role in managing uncertainty (Garrison et al., 2023; Drummond et al., 2023). The “Strategic Mitigation” pathway, where high system adaptability is a core condition for success, empirically substantiates this infrastructural advantage (Garrison et al., 2023; Qiu et al., 2022).

This study also provides robust quantitative evidence for a long-held belief in the rare disease community: the influence of Patient Advocacy Groups (PAGs) is a core determinant of access (Patterson et al., 2023; Sin et al., 2021; Wheeden et al., 2025). The HLM established strong PAG Influence as a statistically significant independent predictor of positive HTA outcomes. To fully understand this influence, it is necessary to disaggregate it into two distinct but complementary mechanisms. The first is the foundational role of qualitative advocacy, where PAGs provide powerful testimony on the lived experience of a disease and the high unmet need, increasing the moral and political pressure on HTA committees to find a path to access (Sin et al., 2021). The second, and rapidly emerging, mechanism is the role of PAGs in quantitative evidence generation through formal Patient Preference (PP) studies and the collection of patient-reported outcomes (Bertelsen et al., 2025; Gentilini and Rana, 2025; May et al., 2025).

The “Strategic Mitigation” pathway from our QCA, in which strong PAG influence is a key condition for overcoming high evidence uncertainty (Ocloo et al., 2021), becomes far more intelligible through this lens. The power of the patient voice in this context likely stems from the synergy of these two mechanisms: qualitative advocacy establishes the urgency to act despite uncertainty, while quantitative preference data provides formal evidence to help HTA committees manage that uncertainty.

The landscape of HTA in Europe is being fundamentally reshaped by the EU HTAR. The regulation’s mandate for Joint Clinical Assessments (JCAs) will centralize and standardize the debate around therapy-level predictors like Clinical Efficacy and Comparative Evidence (Desmet et al., 2024; Sarri et al., 2025). This will shift the locus of access differentiation squarely to the national level, where non-clinical assessments and final reimbursement decisions will be made (Sarri et al., 2025; Merész et al., 2025; Wang and McAuslane, 2025).

Once a JCA report is published, the final decision in each member state will hinge precisely on the nationally variable factors this study has shown to be critical: affordability (driven by Therapy Cost and national GDP), System Adaptability (the national infrastructure for IPMs and RWE), and the influence of local PAGs. Therefore, this study’s model provides an essential roadmap for navigating the post-HTAR access journey. It highlights the factors that will determine whether a positive EU-level clinical assessment translates into actual patient access, informing the dual-level evidence strategies that stakeholders must now develop (Supplementary Appendix D).

Our model provides two distinct types of insight: For non-JCA bodies (NICE, CADTH, etc.): Our findings are a direct, empirical explanation of their current decision-making. For the new EU JCA context: Our findings serve as a powerful predictive model. The JCA will centralize the assessment of therapy-level clinical evidence (Morgese et al., 2025). Our analysis of non-JCA systems provides a “natural experiment” for what happens next. It demonstrates that when clinical assessment is standardized, the differentiating factors for access will shift squarely to the national-level factors we have identified: national affordability (GDP), local System Adaptability (e.g., IPM infrastructure), and local PAG Influence. Therefore, our findings are highly valid for JCA-participating states, as they highlight the specific national hurdles that will become the locus of post-JCA access negotiations.

This study’s strengths are its multi-country scope and dual-methodology design; The HLM was chosen to quantify the independent net effects of variables while correctly accounting for the nesting of HTA decisions within agencies and countries. This avoids the underestimated standard errors and increased risk of Type I errors that would result from using standard regression techniques that ignore data clustering while Fuzzy-Set Qualitative Comparative Analysis (fsQCA) was particularly useful for understanding complex situations where multiple pathways can lead to the same result (equifinality) and where the effect of one factor depends on the presence of others (conjunctural causation). Instead of isolating single variables, fsQCA identifies which specific “bundles” of conditions are necessary or sufficient to produce the outcome you’re studying.

However, several limitations must be acknowledged. The HLM, while statistically appropriate, assumes linear relationships and its coefficients represent “average” effects, which may not fully capture the complexity of agency-specific decision-making—a key reason the QCA was also necessary. The analysis is confined to seven developed nations, and its findings may not be generalizable to health systems with different resource levels or value frameworks. The coding of qualitative data from HTA reports, while systematic, inherently involves a degree of researcher interpretation (Bradley et al., 2007). Unobserved variables, such as the impact of informal negotiations between manufacturers and payers, could not be captured (Wettstein and Boes, 2020; Callenbach et al., 2022). Crucially, this model is built on pre-HTAR data. While it provides a robust baseline for understanding the dynamics of national-level assessment, its predictive validity in the new, bifurcated reimbursement environment is an empirical question that must be tested over time.

This points to several vital avenues for future research. First, this analysis should be replicated in three to 5 years to quantitatively assess how the predictive weights of different factors and the composition of the QCA pathways have shifted after the full implementation of the HTAR. Second, the large-N analysis presented here would be powerfully complemented by in-depth, comparative case studies of the first CGTs to undergo the JCA process. Finally, future research should aim to develop more granular metrics for both System Adaptability and PAG Influence to further test and refine the mechanistic links proposed in this discussion. A key limitation is our focus on PAGs as the primary stakeholder group, which was a pragmatic choice based on data availability in HTA reports. Future research should expand this model to also quantify the influence of other key stakeholders, such as clinical societies and payer groups.

## Conclusion

The central conclusion of this research is that achieving market access for CGTs requires a sophisticated, configurational strategy that integrates clinical evidence and stakeholder advocacy within the specific decision-making context. Our findings reveal two distinct pathways: the ‘Transformative Value’ path, a classic innovation-led strategy, and the ‘Strategic Mitigation’ path, an empirical evidence strategy, where System Adaptability (e.g., HTA bodies acting as “flexible managers of uncertainty”) and strong PAG influence combine to overcome high evidence uncertainty through ecosystem co-evolution. Manufacturers must skillfully select the right pathway based on their evidence and the specific HTA context.

Based on our HLM and QCA findings, we recommend policymakers prioritize investment in enhancing national HTA infrastructure (e.g., data registries, administrative capacity) to improve System Adaptability, enabling effective implementation of IPMs and uncertainty management. Given the post-HTAR landscape, where clinical evidence will be standardized, manufacturers should also develop ‘Strategic Mitigation’ packages, incorporating IPMs and patient-preference data to address uncertainty. This collective adaptation of the ecosystem is essential to realizing the full clinical and societal benefits of these transformative medicines.

## Data Availability

The original contributions presented in the study are included in the article/Supplementary Material, further inquiries can be directed to the corresponding author.
