# Supplementary material for: A hierarchical and configurational analysis of Health Technology Assessment outcomes for cell and gene therapies
Source: Front Pharmacol. 2025 Dec 1;16:1695961. doi: 10.3389/fphar.2025.1695961 (PMC12702701; doi:10.3389/fphar.2025.1695961)
Supplement: Supplementary file 1 [file Supplementaryfile1.docx]

A Hierarchical and Configurational Analysis of Health Technology Assessment Outcomes for Cell and Gene Therapies

Appendix A: Systematic Search Strategy

To construct the comprehensive dataset (as described in L137), a multi-pronged search strategy was implemented to identify all relevant HTA appraisals. This strategy combined (1) a direct, manual search of all selected HTA agency websites, (2) a supplementary search of bibliographic databases, and (3) a "snowballing" search of reference lists.

A.1. Primary Search: HTA Agency Websites

This was the primary strategy for identifying the full cohort of HTA appraisals (the unit of analysis). The public-facing websites and report repositories of the seven selected jurisdictions were manually searched between July 1, 2025, and July 31, 2025.

- **Agencies Searched:** CADTH (Canada), NICE (UK), G-BA (Germany), HAS (France), AIFA (Italy), AEMPS (Spain), PBAC/MSAC (Australia).
- **Search Terms:** Searches were conducted using all known brand and generic names for all EMA- and FDA-approved CGTs as of July 2025. This list included, but was not limited to: 'Kymriah' (tisagenlecleucel), 'Yescarta' (axicabtagene ciloleucel), 'Luxturna' (voretigene neparvovec), 'Zolgensma' (onasemnogene abeparvovec), 'Zynteglo' (betibeglogene autotemcel), 'Tecartus' (brexucabtagene autoleucel), 'Abecma' (idecabtagene vicleucel), 'Carvykti' (ciltacabtagene autoleucel), 'Hemgenix' (etranacogene dezaparvovec), 'Libmeldy' (atidarsagene autotemcel), 'Casgevy' (exagamglogene autotemcel), 'Lyfgenia' (lovotibeglogene autotemcel), 'Roctavian' (valoctocogene roxaparvovec), and 'Upstaza' (eladocagene exuparvovec).
- **Rationale:** This direct search was essential for capturing the complete HTA documentation, including final reports, committee minutes, and stakeholder submissions, which are not indexed in academic databases.

A.2. Secondary Search: Bibliographic Databases

To identify related publications and ensure no major public reports were missed, supplementary searches were conducted in MEDLINE (via PubMed) and the INAHTA database. Table D1 provides the logic and illustrative search strings.

**Table A1: Database Search Strategy and Rationale**

| **Database/Source** | **Search Strategy** | **Rationale** |
| --- | --- | --- |
| **MEDLINE (via Ovid/PubMed)** | ((('Gene Therapy'[Mesh] OR 'Cellular Therapy'[Mesh] OR "advanced therapy medicinal product*".tw. OR "CAR-T".tw. OR "CGT".tw.)) AND (('Technology Assessment, Biomedical'[Mesh] OR "health technology assessment".tw. OR "HTA".tw. OR 'Reimbursement'[Mesh] OR "reimbursement".tw. OR "cost-effectiveness".tw.)) AND (("NICE".tw. OR "CADTH".tw. OR "G-BA".tw. OR "HAS".tw. OR "AIFA".tw. OR "PBAC".tw. OR "AEMPS".tw.))) | To identify peer-reviewed publications, systematic reviews, and grey literature (e.g., conference abstracts) discussing the HTA of CGTs in our target jurisdictions. |
| **INAHTA Database** | Advanced keyword search for: "Gene Therapy", "Cell Therapy", "ATMP", and all specific brand/generic names listed in the primary search. | To cross-reference reports identified in the primary search and to locate any additional assessments from the selected HTA bodies, as INAHTA aggregates reports from its members. |

A.3. Tertiary Search: Snowballing

The reference lists of all included HTA reports and relevant systematic reviews identified in the secondary search were manually scanned to identify any additional appraisals that met the inclusion criteria.

Appendix B: Methodological Note on the Construction of Composite Indicators

B.1. Theoretical Framework

To capture complex, multidimensional concepts not directly reported as single data points in HTA documents, we constructed two composite indicators: System Adaptability and PAG Influence. The construction process was guided by best practices for developing composite indicators, emphasizing theoretical grounding, transparency, and robustness, as outlined by the OECD and others. The goal was to create quantifiable measures that reflect the underlying latent concepts of an HTA system's flexibility and the substantive impact of patient engagement.

B.2. Construction of the 'System Adaptability' Indicator

System Adaptability is defined as an HTA agency's structural capacity and demonstrated experience in accommodating the unique challenges of CGTs. The composite score (ranging from 1 to 5) was created by summing the scores of three equally weighted sub-indicators, each coded based on publicly available agency methodology guides, policy documents, and HTA reports.

| Sub-Indicator | Definition & Coding | Data Source(s) |
| --- | --- | --- |
| Dedicated Flexible Pathway | Existence of a formal, specialized pathway for high-cost, high-need, or highly specialized technologies. | Agency methodology guides (e.g., NICE HST Programme documents ^39^) |
|  | 0 = No dedicated pathway. |  |
|  | 1 = Dedicated pathway exists. |  |
| Documented Use of Innovative Payment Models (IPMs) | Evidence of the HTA system having implemented outcomes-based or amortized payment models for any therapy. | HTA reports, policy documents, literature (e.g., AIFA's use of "Payment at Results" ^12^) |
|  | 0 = No documented use of IPMs. |  |
|  | 1 = IPMs discussed but not implemented. |  |
|  | 2 = At least one IPM documented and implemented. |  |
| Formal Real-World Evidence (RWE) Framework | Existence of an established, formal process for using RWE to resolve uncertainty, often via Managed Entry Agreements (MEAs). | Agency methodology guides, policy documents ^1^ |
|  | 0 = No formal RWE framework. |  |
|  | 1 = RWE used ad-hoc. |  |
|  | 2 = Formal framework for Coverage with Evidence Development or similar MEAs exists. |  |

B.3. Construction of the 'PAG Influence' Indicator

PAG Influence is defined as the documented level of engagement and substantive impact of Patient Advocacy Groups in a specific HTA review. The ordinal score (ranging from 0 to 3) was coded based on a hierarchical assessment of patient involvement as documented in the final HTA reports and supporting materials.

| Score | Level of Influence | Definition & Coding Criteria | Data Source(s) |
| --- | --- | --- | --- |
| 0 | No/Minimal Influence | No PAG submission is mentioned in the HTA documentation, or only a boilerplate letter of support is provided. | HTA reports, committee minutes |
| 1 | Passive Input | A formal written submission from a PAG is documented and cited, providing patient perspectives on disease burden and unmet need. | HTA reports, patient group submissions |
| 2 | Active Participation | In addition to a written submission, a patient expert or PAG representative is documented as having presented testimony or participated actively in committee meetings/hearings. | HTA reports, committee minutes |
| 3 | Evidence Co-Creation | In addition to active participation, the HTA report explicitly references or uses patient-generated evidence (e.g., a formal patient preference study, a PAG-led survey on quality of life) as a key input for its deliberations. | HTA reports, economic modeling reports |

B.4. Validation and Sensitivity Analysis

The internal consistency of the composite indicators was assessed. For the HLM analysis, sensitivity analyses were conducted using alternative weighting schemes for the sub-indicators to ensure the robustness of the main findings. The results remained stable, supporting the use of the primary scoring methodology.

Appendix C: QCA Calibration Anchors

Calibration is the process of assigning set membership scores to cases. For this fsQCA, we used the direct calibration method, defining three anchors for each condition based on theoretical knowledge and the empirical distribution of the data: full membership (fuzzy score = 1), the crossover point of maximum ambiguity (fuzzy score = 0.5), and full non-membership (fuzzy score = 0).

| Condition | Raw Data Format | Full Non-Membership (0) | Crossover Point (0.5) | Full Membership (1) | Justification |
| --- | --- | --- | --- | --- | --- |
| HTA Outcome | Ordinal (-2 to +2) | ≤ -1 | 0 | ≥ +1 | Positive outcome defined as at least a straightforward recommendation. |
| Clinical Efficacy | Ordinal (-1 to +1) | -1 | 0 | 1 | High efficacy defined as superiority on a final clinical outcome. |
| Therapy Cost | Continuous ($USD) | < $1,000,000 | $2,500,000 | > $4,000,000 | Anchors reflect the range of recent CGT list prices. ^43^ |
| Unmet Need | Composite (1-5) | 1 | 3 | 5 | Based on the distribution of the composite score. |
| System Adaptability | Composite (1-5) | 1 | 3 | 5 | Based on the distribution of the composite score. |
| PAG Influence | Ordinal (0-3) | 0 | 1.5 | 3 | High influence defined as active participation or evidence co-creation. |
| High GDP | Continuous ($USD) | < $45,000 | $60,000 | > $75,000 | Anchors based on World Bank classifications for high-income economies. |
| High Uncertainty | Binary (0/1) | 0 | 0.5 | 1 | Dichotomous condition based on HTA report's explicit statement of high uncertainty regarding durability or surrogate validity. |
| Rare Disease | Binary (0/1) | 0 | 0.5 | 1 | Based on formal orphan drug designation. |
| Cost-Saving | Binary (0/1) | 0 | 0.5 | 1 | Based on manufacturer's claim and HTA's economic evaluation. |

Appendix D: Matrix of Strategic Recommendations for Stakeholders in the Post-HTAR Era

| **Stakeholder** | **Key Finding from this Study** | **Strategic Recommendation for the Post-HTAR Era** |
| --- | --- | --- |
| **Manufacturers** | Equifinality & Configurational Logic: Multiple pathways to success exist (e.g., "Transformative Value," "Strategic Mitigation"). | Develop a Dual-Level Evidence Strategy: Prepare a robust clinical dossier for the EU-level JCA, but simultaneously develop a tailored *national-level value proposition* focusing on budget impact models, IPM feasibility, and local patient needs to address the post-JCA hurdles identified in this study. |
|  | The Power of the Patient Voice: Strong PAG influence is a key enabler, especially under evidence uncertainty. | Evolve PAG Engagement from Support to Partnership: Move beyond simple grants. Co-develop evidence with PAGs, including qualitative burden-of-illness studies for the JCA narrative and quantitative Patient Preference studies to justify endpoints and risk-benefit trade-offs. |
| **HTA Agencies & Policymakers** | System Adaptability as a Critical Enabler: The capacity to manage uncertainty via IPMs and RWE is a key determinant of access. | Invest in National Infrastructure to Close the "Adaptability Gap": The JCA standardizes clinical review; national differentiation will depend on the ability to manage cost and uncertainty. Prioritize investment in data registries and administrative capacity to implement IPMs effectively. |
|  | Asymmetric Causation & Veto Configurations: High uncertainty, high cost, and low adaptability is a potent recipe for failure. | Formalize National Processes for JCA Integration: Develop clear, transparent national pathways for how JCA reports will be used in local economic evaluations. This prevents the JCA from becoming a "fifth hurdle" and ensures a structured process for therapies with positive clinical assessments. |
| **Patient Advocacy Groups (PAGs)** | PAGs as Core Determinants: PAG influence is not a "soft" factor but a statistically significant predictor of positive outcomes. | Professionalize Evidence Generation Capabilities: Build internal or partnered expertise in methodologies like Patient Preference studies. Prepare to engage formally in the JCA's stakeholder consultation process and provide robust, evidence-based input at both the EU and national levels. |
